# Supplementary material for: Recruitment and Retention Strategies Used in Dietary Randomized Controlled Interventions with Cancer Survivors: A Systematic Review
Source: Cancers (Basel). 2023 Sep 1;15(17):4366. doi: 10.3390/cancers15174366 (PMC10486591; doi:10.3390/cancers15174366)
Supplement: Supplementary file 1 [file cancers-15-04366-s001.zip › cancers-2587293-supplementary.pdf]

Supplementary Figure S1. Search Terms

Filters applied to the search include randomized controlled trials, controlled clinical trials, adults, humans, English, and published 2013-2023.

Breast Cancer	Colorectal Cancer	Prostate Cancer	Lung Cancer
breast cancer*<br>breast neoplasm*	colorectal cancer*<br>colorectal neoplasm*	prostate cancer*<br>prostate neoplasm*	lung cancer*<br>lung neoplasm*

AND

Survivor	
survivor*<br>patient*	cancer survivor*<br>participant

AND

Randomized Controlled Trial/Clinical Trial		
"randomized controlled trial"<br>randomized	"controlled trial"<br>"clinical controlled trial"	"clinical trial"<br>"controlled clinical trial"

AND

Diet		
diet*<br>nutrition<br>"diet therapy"	"nutrition counseling"<br>vegetarian<br>"diet intervention"	Mediterranean<br>"Diet, Food, and Nutrition"<br>"nutrition therapy"

AND

Recruitment/Retention		
recruit<br>recruitment<br>screen<br>eligible	ineligible<br>retain<br>retention	attrition<br>dropout<br>withdraw
